# Biomimetic ion substituted and Co-substituted hydroxyapatite nanoparticle synthesis using *Serratia Marcescens*

**DOI:** 10.1038/s41598-023-30996-z

**Published:** 2023-03-18

**Authors:** Mareeswari Paramasivan, T. S. Sampath Kumar, Hemalatha Kanniyappan, Vignesh Muthuvijayan, T. S. Chandra

**Affiliations:** 1 Department of Biotechnology, Indian Institute of Technology Madras, Chennai, Tamil Nadu 600036 India; 2 Medical Materials Laboratory, Department of Metallurgical and Materials Engineering, Indian Institute of Technology Madras, Chennai, Tamil Nadu 600036 India

**Keywords:** Biomaterials, Biomimetics, Biomaterials, Nanoscale materials, Nanobiotechnology, Nanoscale materials

## Abstract

Biomimicry is becoming deep-rooted as part of bioceramics owing to its numerous functional advantages. Naturally occurring hydroxyapatite (HA) apart from primary nano structures are also characterised by various ionic substitutions. The ease of accommodating such key elements into the HA lattice is known to enhance bone healing properties of bioceramics. In this work, hydroxyapatite synthesized via biomimetic approach was substituted with individual as well as multiple cations for potential applications in bone repair. Ion substitutions of Sr, Mg and Zn was carried out on HA for the first time by using *Serratia* grown in a defined biomineralization medium. The individual ions of varying concentration substituted in *Serratia* HA (SHA) (Sr SHA, Mg SHA and Zn SHA) were analysed for crystallinity, functional groups, morphology and crystal size. All three showed decreased crystallinity, phase purity, large agglomerated aggregates and needle-shaped morphologies. Fourier transform infrared spectroscopy (FTIR) spectra indicated increased carbonate content of 5.8% resembling that of natural bone. Additionally, the reduced O–H intensities clearly portrayed disruption of HA lattice and subsequent ion-substitution. The novelty of this study lies primarily in investigating the co-substitution of a combination of 1% Sr, Zn and Mg in SHA and establishing the associated change in bone parameters. Scanning electron microscope (SEM) and transmission electron microscope (TEM) images clearly illustrated uniform nano-sized agglomerates of average dimensions of 20–50 nm length and 8–15 nm width for Sr SHA; 10–40 nm length and 8–10 nm width for both Zn SHA and Mg SHA and 40–70 nm length and 4–10 nm width in the case of 1% Sr, Zn, Mg SHA. In both individual as well as co-substitutions, significant peak shifts were not observed possibly due to the lower concentrations. However, cell volumes increased in both cases due to presence of Sr^2+^ validating its dominant integration into the SHA lattice. Rich trace ion deposition was presented by energy dispersive X-ray spectroscopy (EDS) and quantified using inductively coupled plasma optical emission spectrometer (ICP-OES). In vitro cytotoxicity studies in three cell lines viz. NIH/3T3 fibroblast cells, MG-63 osteosarcoma cells and RAW 264.7 macrophages showed more than 90% cell viability proving the biocompatible nature of 1% Sr, Zn and Mg in SHA. Microbial biomineralization by *Serratia* produced nanocrystals of HA that mimicked “bone-like apatite” as evidenced by pure phase, carbonated groups, reduced crystallinity, nano agglomerates, variations in cell parameters, rich ion deposition and non-toxic nature. Therefore ion-substituted and co-substituted biomineralized nano SHA appears to be a suitable candidate for applications in biomedicine addressing bone injuries and aiding regeneration as a result of its characteristics close to that of the human bone.

## Introduction

One of the most important materials among the family of calcium phosphates is HA. It dominates the medical industry with extensive applications as a result of its exceptional biocompatibility properties, high resorption and slow dissolution rates in the body^1^. Although the inherent elemental compositions of HA resemble the mineral components of teeth and bones; they depict poor mechanical strength and fatigue behaviour. This comes as a consequence of coarser grain sized HA as opposed to nano HA, restricting its utilization in non-load bearing applications. Despite, excellent conductivity and biocompatibility, such limitations of low toughness and brittleness contributes to unreliability^2^. Currently, clinical and experimental research revolves around developing bioceramics characterized by biomimicry approaches to overcome such limitations and enhance biological performance as well. In particular, the substitution of various ions into the nano structure of HA with a view of altering its biocompatibility, sinterability and mechanical properties reflects growing interest^1^. As, the uniqueness of biological apatite is the associated presence of a number of foreign ions that contribute effectively in bone repair based on the dosage. Biological apatite differs from stoichiometric HA by the prevalence of various amounts of vicarious ions either incorporated or adsorbed onto its apatite lattice, including anionic (F^−^, Cl^−^, SiO_4_^4−^ and CO_2_^3−^) and/or cationic substitutions (Na^+^, Mg^2+^, K^+^, Sr^2+^, Zn^2+^, Ba^2+^, Al^3+^)^3^. Earlier, fracture toughness of pure HA was improved by cationic substitutions from a range of 0.5–1 MPa m^1/2^ to 2.7 MPa m^1/2^ with 0.6 wt% Mg and 1.5 MPa m^1/2^ at 5 at.% Zn. Furthermore, such cationic substitutions increased bioactivity, surface reactivity and showed excellent cell proliferation capabilities^4^. The doping of such ions as multi-ion substitutions or co-substitutions has also been reported producing multi-faceted attributes^5,6^.

It is well known that HA lattice owing to its high structural flexibility can accommodate within its lattice, nearly half of the elements in the periodic system; allowing substitutions of diverse vicarious ions for its three sublattices namely Ca^2+^, PO_4_^3−^ and OH^−^ groups. As a consequence, such incorporations affect the physiochemical and biological properties of HA. In terms of crystal structure, crystallinity, thermal stability, surface charge, solubility, in vitro bioactivity, osteoclastic and osteoblastic responsiveness in vitro and bone regeneration in vivo^3^. Both bivalent (e.g., Sr^2+^, Ba^2+^, Mg^2+^, …) and monovalent cations (e.g., Na^+^, K^+^, …) predominantly substitute Ca^2+^ sites. The presence of these cations in bones contribute greatly in the formation and reabsorption of the bone. For instance, Mg^2+^ ion deficiency declines the growth rate of osteoblasts causing reduction in bone mass density. Whereas, Zn^2+^ ions enhance bone formation by accelerating growth rate and Sr^2+^ is non-toxic even in large doses in the human body and known to poses dual trait of promoting bone formation and reducing of bone resorption^6–8^. Literature highlights improved biological performance of such ion-substituted HA materials^9^. Sr HA besides preventing bone resorption and improving mechanical properties also depicted higher volumes of new bone formation in vivo rabbit models. Similarly, Zn HA also showed enhanced new bone formation when implanted in vivo rat and rabbit models for one and two months, respectively. While, in vivo tests on New Zealand White rabbits wherein Mg (15 at.%) was deployed as filling for femoral bone defects, portrayed increased osteoconductivity^4^.

Indeed, the presence of such ions in the bones are known to act as buffering agents in the bloodstream and directly influence the proliferation of both osteoblasts and osteoclasts^8^. More interestingly, some ions (e.g., Sr^2+^ and Zn^2+^) reduces the crystallinity of HA, thereby improving dissolution while the others (e.g., F^−^) enhances the crystallinity and subsequently reduces dissolution^10^. Besides, Sr^2+^ and/or Zn^2+^ is known to offset any cytotoxicity that arises due to high concentrations of Ag ions^11^. Further, enhanced host bone healing without undesirable bacterial infections and inflammatory responses was observed in Zn^2+^ and Mg^2+^ co-doped nHA structures^12^. Recently, a new bone cement of co-substituted Sr^2+^ and Mg^2+^ (with 5 mol% of Sr and 5 mol% of Mg) nano-hydroxyapatite (n-HAs) with calcium phosphate dibasic and chitosan/gelatin polymers showed exceptional osteoblast activity for bone regeneration^13^. Such co-substitutions of ions thus offer conducive environments by combining several desirable characteristics than individual ion-substitutions of HA. However, the selection of ions for co-substitution needs to be carefully considered with respect to atomic substitution sites, phase stability, biological impact and ionic charge equilibrium^14^.

In the present study, with a view of further enhancing the properties and symmetry of synthesized HA, ionic substitutions was investigated for the first time in *Serratia sp*. biomineralized HA adopting a biomimetic method of synthesis^15^. Both individual and co-substitution of cations was studied and characterised for its competence in terms of functional groups, crystallinity, morphology and biocompatibility. Bacterial synthesized HA was co-substituted with a combination of Sr, Zn and Mg ions with an aim of mimicking the biological apatite and to comprehend the advantageous traits for applications in bone repair. Study of such ionic substitutions would potentially aid a better understanding of the biomineralization processes to control the properties of the precipitated phase, increase bioactivity and the delivery of ions for treating bone diseases^16^.

## Materials and methods

All chemical reagents used were of analytical grade. Calcium chloride (CaCl_2_), Strontium chloride (SrCl_2_), Zinc chloride (ZnCl_2_), Magnesium chloride (MgCl_2_), Trisodium citrate, tris buffer, and toluene were procured from SRL chemicals, India. *β*-glycerophosphate disodium salt dihydrate, para-nitrophenyl phosphate (p-NPP) were procured from Sigma-Aldrich (India). Nutrient Broth, Dulbecco’s modified eagle medium (DMEM), Minimum Essential Medium (MEM), Antibiotic penicillin–streptomycin, Fetal Bovine Serum (FBS) were purchased from Himedia, India.

### Synthesis of HA using a Gram-negative bacteria *Serratia*

*Serratia marcescens* was grown in 100 ml of nutrient broth medium in conical flask incubated at 30 °C for 15 h. Cells were collected by centrifugation at 3500 rpm for 15 min and washed with 0.85% NaCl. This cell suspension was added to a mineralization media consisting of 10 mM calcium chloride, 25 mM *β*-glycerophosphate disodium salt dihydrate, 20 mM trisodium citrate, and 25 mM tris buffer, with pH adjusted to 8.5^17^. After incubation of *Serratia* in mineralization medium for 10 days, the white precipitated material was collected and dried at 100 °C for 7 h.

### Synthesis of strontium (Sr) or zinc (Zn) or magnesium (Mg) substituted HA nanoparticles

Calcium ions in mineralization media was substituted with Sr, Zn or Mg ions to obtain the corresponding ion substituted HA. It was vital to ensure that the concentration of ions in the media, in no way affected the bacteria culture conditions. *Serratia* carried out synthesis of HA under conditions stated earlier in "Synthesis of HA using a Gram-negative bacteria *Serratia*" Section. CaCl_2_ in the mineralization media was replaced by 3, 5 and 10% SrCl_2_ and the precipitate collected was designated as 3% Sr SHA, 5% Sr SHA and 10% Sr SHA. Similarly, 2, 4 and 6% ZnCl_2_ was substituted for CaCl_2_ in the mineralization media and the corresponding 2% Zn SHA, 4% Zn SHA and 6% Zn SHA precipitates were collected. Likewise, 2, 4 and 6% MgCl_2_ was substituted for CaCl_2_. However, precipitation of HA by *Serratia* only occurred at 2% MgCl_2_. The precipitate 2% Mg SHA was collected, dried at 100 °C for 7 h and analyzed.

### Synthesis of Co-substituted HA

To develop an apatite similar to bone mineral, co-substitution of ions was performed. All three ions (Sr, Mg, Zn) were replaced together in the mineralization media as described in "Synthesis of HA using a Gram-negative bacteria *Serratia*" Section by 1% of SrCl_2_, MgCl_2_, ZnCl_2_ and further synthesis of HA by *Serratia* was carried out. Precipitation of HA by *Serratia* occurred only at 1% of SrCl_2_, MgCl_2_, ZnCl_2_ concentration in the media. The powder collected 1% Sr, Mg, Zn SHA nanoparticles was dried at 100 °C for 7 h and analyzed.

### Characterization of synthesized ion substituted nanoparticles

All individual ion-substituted nanoparticles and co-substituted nanoparticles were characterized using FTIR, XRD, SEM, TEM and ICP-OES techniques. Functional group of the samples were analyzed using FTIR (Perkin—Elmer Spectrum Two, USA) in the range of 500–4000 cm^−1^, with a resolution of 4 cm^−1^. The diffraction patterns of the sample were recorded by XRD (Bruker D8 discover powder XRD, Germany) using Cu/Kα radiation (*λ* = 1.54 Å) at a scanning rate of 1 step/s with a step size of 0.10. The cell volume and cell parameters were calculated from the XRD data using the program “UnitCell”^18^. Surface morphology and the elemental composition of the samples were examined by SEM fitted with EDS (FEI Quanta FEG 200, Netherland) after carrying out gold sputtering and operated at an accelerating voltage of 10 kV. The morphology and particle size of the crystals were determined by TEM operated at 120 keV (Philips CM20 TEM, Netherlands). TEM specimens were prepared by dropping nanoparticles dispersed in ethanol over a carbon-coated copper grid. The quantitative measure of several ions in the samples was evaluated using ICP-OES Perkin Elmer Optima 5300 DV, USA. 10–15 mg of powdered sample was dissolved in 3 ml of 1 M nitric acid and 27 ml of distilled water and the total 30 ml solution was subjected to ICP-OES analysis.

### Biocompatibility evaluation

Cytotoxicity of the prepared nanoparticles was determined by MTT assay with three different cell lines namely NIH/3T3 fibroblast cells, MG-63 osteosarcoma cells, and RAW 264.7 macrophages^15,19,20^. The cell lines were cultured to a cell density of 1 × 10^4^ per well in 96 well plates and incubated with cell culture medium for 24 hours^21^. Once the cell monolayer was formed, the cells were treated with nanoparticles of concentration 100 μg μL^−1^ and then incubated for 24 h. After 24 h, the media was removed and washed with PBS, then each well medium was replaced with 100 μl of 0.5 mg mL^−1^ of MTT [3-(4, 5-dimethylthiazole-2-yl)-2, 5-diphenyl tetrazolium bromide] solution and further incubated for 3 h. Subsequently, the MTT solution was replaced with 200 μl of DMSO (Dimethyl sulphoxide). The optical density (OD) of the wells was measured at 570 nm using a plate reader (Enspire, Perkin Elmer, USA)^22^. The percentage of cell viability in the samples was calculated relative to control using the following equation:

$$\% Cell\;viability = 100 \times \frac{OD\;of\;sample}{{OD\,of\;control}}$$

## Results and discussion

### Individual and co-substituted SHA

Varying concentration range of ions studied were selected based on prior literature^12,23^. In case of Sr and Zn substitutions on SHA, successful deposition was achieved on all three concentrations. However, only a lower concentration of 2% Mg supported HA deposition by *Serratia*. This is in line with the well-established inhibitory effect of Mg on HA nucleation and growth^24,25^. Mg substitution for Ca occurs only over a limited composition and in terms of bacterial deposition 2% was the threshold. Co-substitution was possible only with 1% of SrCl_2_, MgCl_2_, ZnCl_2_ concentration in the mineralization media.

### FTIR analysis

**Figure 1:**
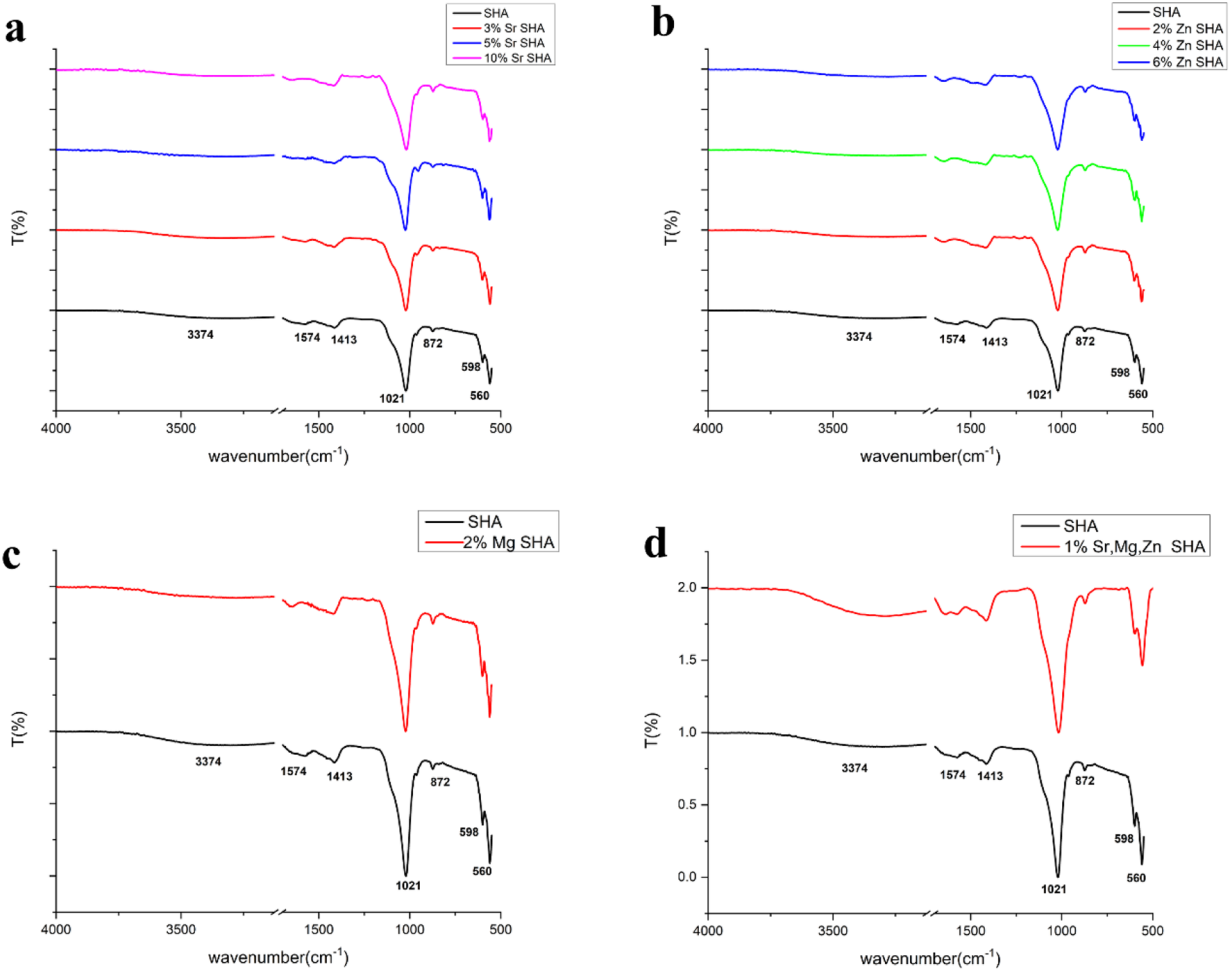
FTIR spectra of (**a**) Sr, (**b**) Zn, (**c**) Mg SHA and (**d**) 1% Sr, Mg, Zn SHA.

FTIR spectra of all the three ion substitutions and co-substitution (Fig. 1a, b, c, d) at varying concentrations displayed the presence of characteristic HA groups similar to SHA spectra. The presence of peaks at 560, 598 cm^−1^ (*ν*4) of O–P–O bending vibrations and 1021 cm^−1^ (*ν*3) of P-O were indicative of phosphate groups corresponding to HA. These results were in alignment with prior reports on Sr, Zn and Mg substitutions^26,27^. In the case of 1% Sr, Mg, Zn co-substituted SHA the most intense band of asymmetric stretching *ν*3, lower intensity bending modes of asymmetric *ν*4 doublet and faint symmetric stretching *ν*1 mode was witnessed, in line with prior results^28^.

Moreover, signature carbonate band at 1413 cm^−1^ (*ν*3—stretching mode) was ascribed to partially substituted phosphate groups in the HA lattice in all four cases. While, the strong band at 872 cm^−1^ (*ν*2- bending mode) of HPO_4_^2−^ overlapping with CO_3_^2−^ clearly evidences ion substitutions. Furthermore, the characteristic vibration of A-type HA, commonly observed at the wavelengths of 877–880 cm^−1^, 1500 cm^−1^ and 1540–1550 cm^−1^, and the AB-type HA (at 1515 cm^−1^) were not observed^29^. Thereby, confirming B-type carbonation in all ionic substitutions of SHA. Such B-type carbonated apatite has been suggested as an effective bone substitute material owing to bioresorbable nature, induction of osteoblast responses and capacity to enhance osteoblast differentiation as well^30^.The amount of CO_3_^2−^ was found to 5.8% in the case of co-substituted SHA and this is known to improve the bioactivity of HA owing to its resemblance with carbonated bone mineral phase.

Percentage of CO_3_^2−^ substitution was calculated using the formula

$${\text{Wt}}.\% {\text{ CO}}_{{3}} = \, \left( {{28}.{62 } \times {\text{ r}}_{{{\text{c}}/{\text{p}}}} } \right) + 0.0{843}$$

where r _c/p_ is the area ratio between the CO_3_^2−^ band (1570–1330 cm^−1^) and the PO_4_^3−^ band (1230–900 cm^−1^)^31,32^.

Thus, the loss of OH^−^ ions from the unit cell evidenced the apparent substitution of Sr, Zn, Mg ions into the lattice of SHA. Previously, decreased intensities of OH^-^ vibration modes were reported for Mg substituted HA at 630 cm^−1^ and 3570 cm^−1^^33^. Similarly in the case of Zn substituted HA the stretching vibration modes of OH^–^ were reported to be lower, as a result of the hydrogen-bonding created by oxygen between OH^–^ and PO_4_^3–^^34^.

It is also worthy to note that literature indicated increasing concentrations of ions corresponds to decreased crystallinity. However, in the present study such broadening of peaks was not presented in any of the four cases, owing to lower concentrations studied. Finally, the broad band at 3374 cm^–1^ corresponds to H_2_O adsorbed on the surface^35^.

### XRD analysis

**Figure 2:**
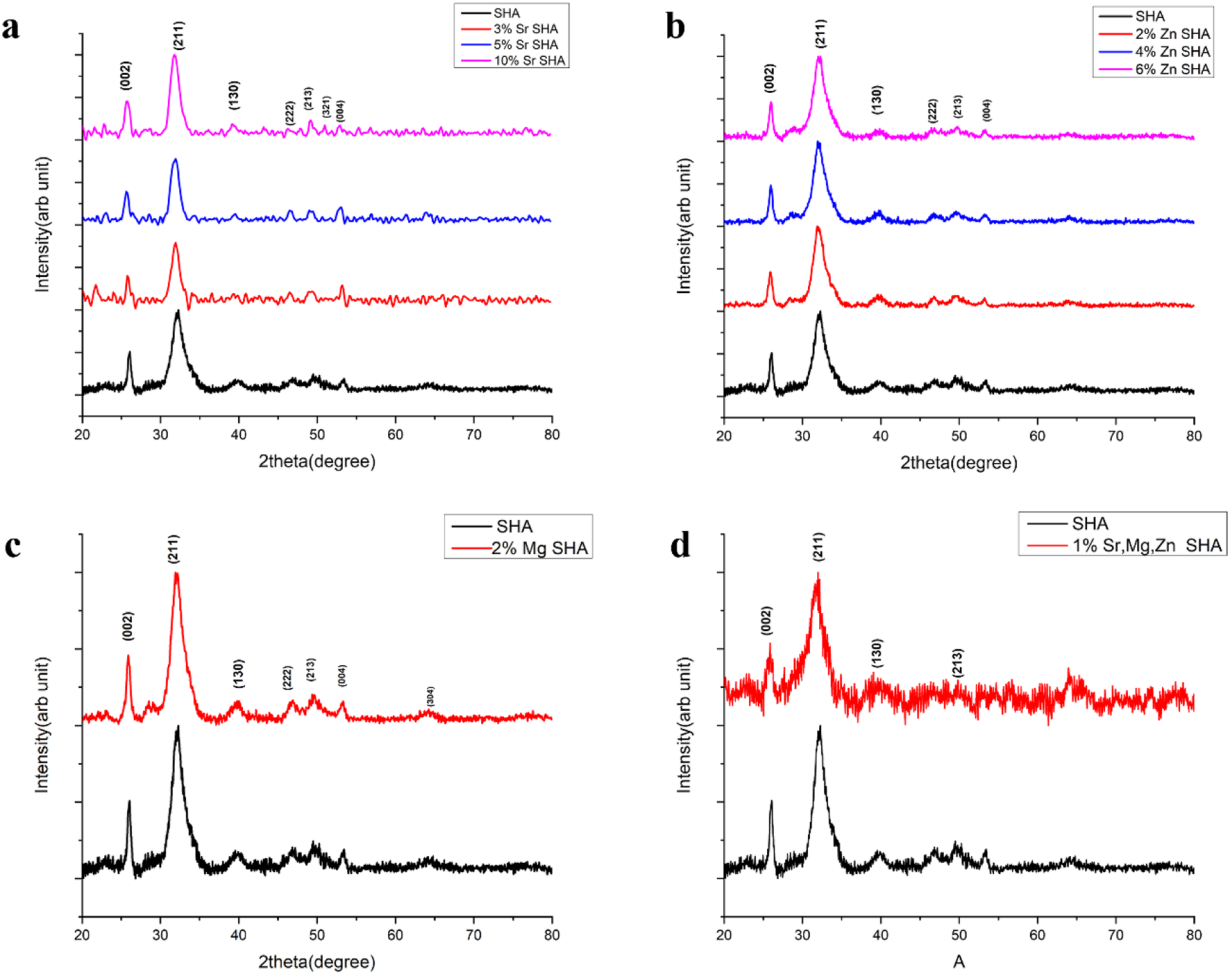
XRD spectra of (**a**) Sr, (**b**) Zn, (**c**) Mg SHA (**d**) 1% Sr, Zn, Mg SHA.

XRD patterns of SHA and ion-substituted HA powders were compared (Fig. 2a, b, c and d) and in all cases reflections associated to HA correlated with reference JCPDS 09-0432. Characteristic peaks representative of lower degree of crystallinity of HA was witnessed. There was no evidence of any secondary phases such as monetite or brushite thus depicting the products obtained were all phase pure^5^. It has been reported that increase in broadening of peaks directly relates to the reduction in crystal size. Such poorly resolved peaks are descriptive of nanoscale (≤ 100 nm) nature and appreciable levels of substitutions within the HA lattice^26^. Besides, no discernible shifts in the distinctive HA peaks were recorded, likely due to lower ion concentrations studied^5,6^.

With respect to crystallinity, reports have discussed the influence of ions on cell volume and lattice parameters. Table 1 depicts the lattice parameters, a and c, and the hexagonal volume of the unit cells of the all samples (Fig. 2a, b, c and d). Strontium displayed a greater preference for Ca (II) site arranged at the apexes of ‘‘staggered’’ equilateral triangles than a Ca (I) of HA structure. As a result of its higher ionic radius greater than that of calcium easily accommodated in Ca (II). This occupancy preference might be responsible for reduced crystallinity. Additionally, validated by the enlargement of the unit cell from 526.46 to 540.15 Å^3^^36^. In contrast, Zn SHA and Mg SHA revealed decreased cell volumes when compared to Sr SHA with values from 526.46 to 524.1 Å^3^ in the case of 2% Mg SHA and to 517.79 Å^3^ in the case of 6% Zn SHA, in line with prior reports^33^.

**Table 1:** Lattice parameters of substituted HA as compared to SHA.

Sample name	Lattice parameter		Cell volume
	a	c	
SHA	9.41	6.86	526.46
3%Sr SHA	9.47	6.84	532.9
5%Sr SHA	9.46	6.94	537.9
10%Sr SHA	9.49	6.91	540.15
2%Zn SHA	9.419	6.86	527.82
4%Zn SHA	9.44	6.83	528.02
6%Zn SHA	9.32	6.87	517.79
2%Mg SHA	9.38	6.87	524.14
1% Sr, Zn, Mg SHA	9.39	6.94	531.07

Previously, Matsunaga et al. established the ion exchange ability of divalent cations with HA via first principle calculations. It was clearly detailed that the ion exchange ability was dependent on the ionic sizes. For the smaller-sized cations of Ni^2+^, Mg^2+^, Cu^2+^, and Zn^2+^, the first nearest neighboring (NN) oxygen ions showed preference to Ca (I) site. While, in the cases of the larger-sized cations Sr^2+^, Pb^2+^, Ba^2+^ at the Ca (I) site, the first NN oxygen ions were seen to displace their positions away from the Ca (I) site^37^.

Along these lines, substitution for Ca^2+^ with Mg^2+^ makes the unit cell smaller, owing to smaller ionic radius of Mg^2+^ (i.e., 0.69 vs. 0.99 Å) and causes a crystallinity decrease due to crystal lattice distortion. Similarly, the substitution of the smaller Zn^2+^ ion for the Ca^2+^ ion within the HA crystal lattice, causes a shrinkage in unit cell parameters^38^. Essentially, the difference between the ionic radii of Sr^2+^ and Ca^2+^ is lesser than the difference between Zn^2+^ and Mg^2+^ with Ca^2+^. Thus, it can be ascribed to the increase in cell volume up to 531.07 Å^3^ in the case of 1% Sr, Zn, Mg SHA. Notably Sr with 10% Sr SHA substitution was found to reflect highest cell volume as mentioned earlier, exhibiting dominance of its substitution in the SHA lattice both individually as well as in co-substituted forms. It is also well known that magnesium ions destabilise the HA structure, promoting the formation of *β-*TCP (Tricalcium phosphate) whereas strontium ions are able to easily substitute within HAp lattice, retaining a single HAp phase^33^. Efficient balance of substitution is thus realized owing to Sr addition incurring phase purity and stabilizing effect.

In terms of lattice parameters, a change in “a” and “c” is ideal for substituted HA. However, the extent of change in “a” and “c” is dependent on the type of substituent and the amount of substitution (Table 1). Detailed effects of substitutions on lattice parameters and the inclined solubility of HA have been illustrated previously^9^. Herein, the refinement recorded that the lattice parameters, a and c, decreased with Zn substitution up to 6% and was also consistent with observations by Li et al.^34^. However, irregular trend depiction in “c” lattice of Zn SHA was noted. Earlier reports have reported decreased values with increasing Zn concentrations^39^. However, some reports have also attributed an observed increase in lattice parameter “c” with an occupancy of new position at hexagonal axis 2b site where Zn pushes the close-by O4 (hydroxyl-O) atoms apart, expanding the c direction and distorting the nearby phosphate tetrahedron by attracting O3 (phosphate-O) atoms and shrinking the “a” direction^40^ or a simple substitution of OH^-^ groups by H_2_O molecules^39^.

Previously, Rajendran et al. highlighted that at low and moderate concentrations, co-substitution of Mg^2+^/Zn^2+^/Co^2+^ ions led to a decrease in basal “a” lattice value and axial “c” lattice parameter slightly increased^41^. Similar results were obtained in the case of co-substitution with decrease in “a” to 9.39 Å and slight increase in “c” to 6.94 Å. It is thus evident that co-substitution remarkably influences the HA features in terms of crystallinity, crystal size, lattice parameters and unit cell volume. Depending on the substituting ions selected, the values gradually decrease or increase. The decrease in crystallinity and particle size as marked in the current study shall likely contribute to increase in dissolution rate thereby enhancing solubility and bio-functionality^42^.

### SEM and EDS analysis

**Figure 3:**
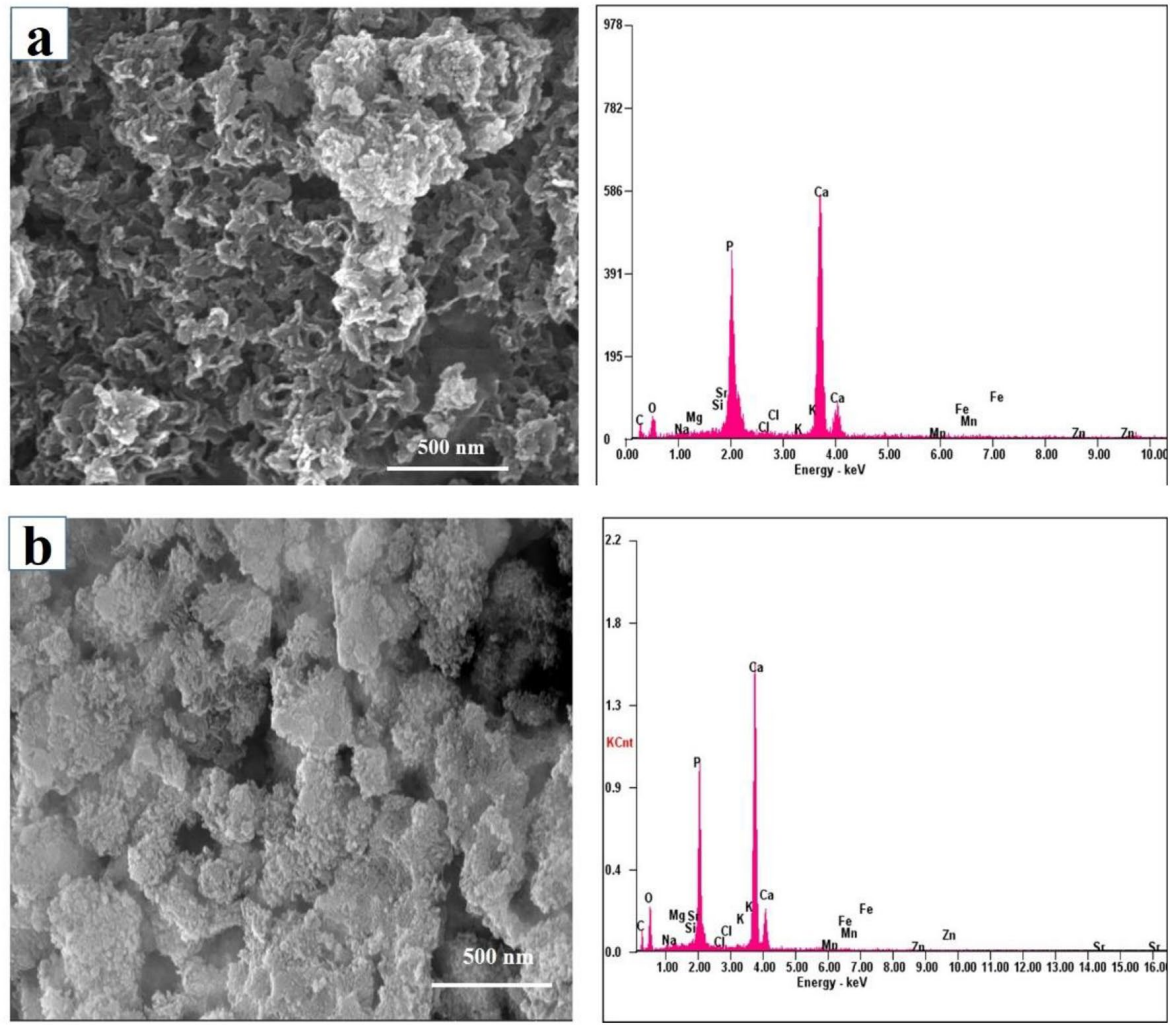
SEM–EDS analysis of (**a**) SHA (**b**) 2%Mg SHA.

**Figure 4:**
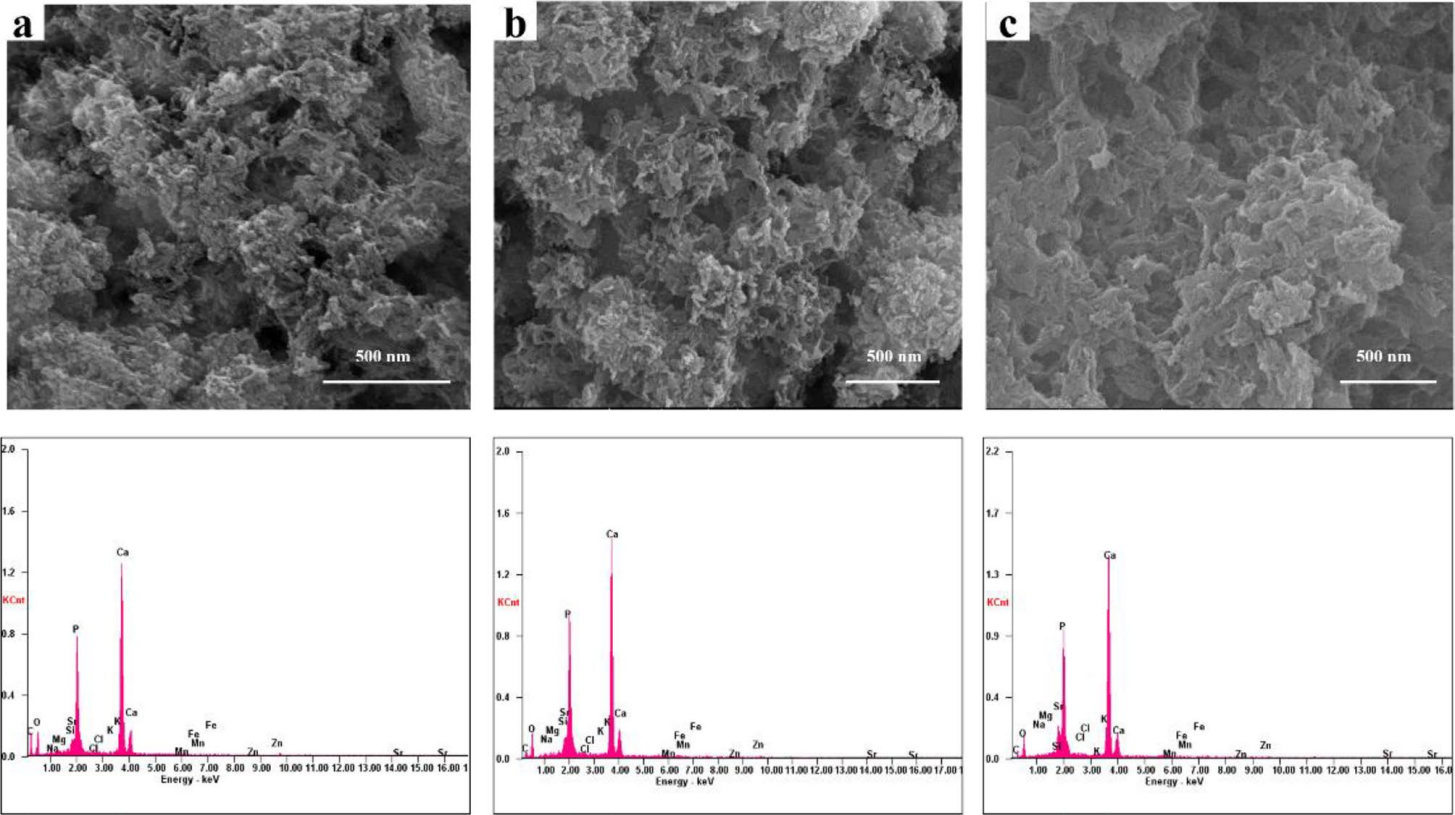
SEM–EDS analysis of (**a**) 3% Sr (**b**) 5% Sr (**c**) 10% Sr SHA.

**Figure 5:**
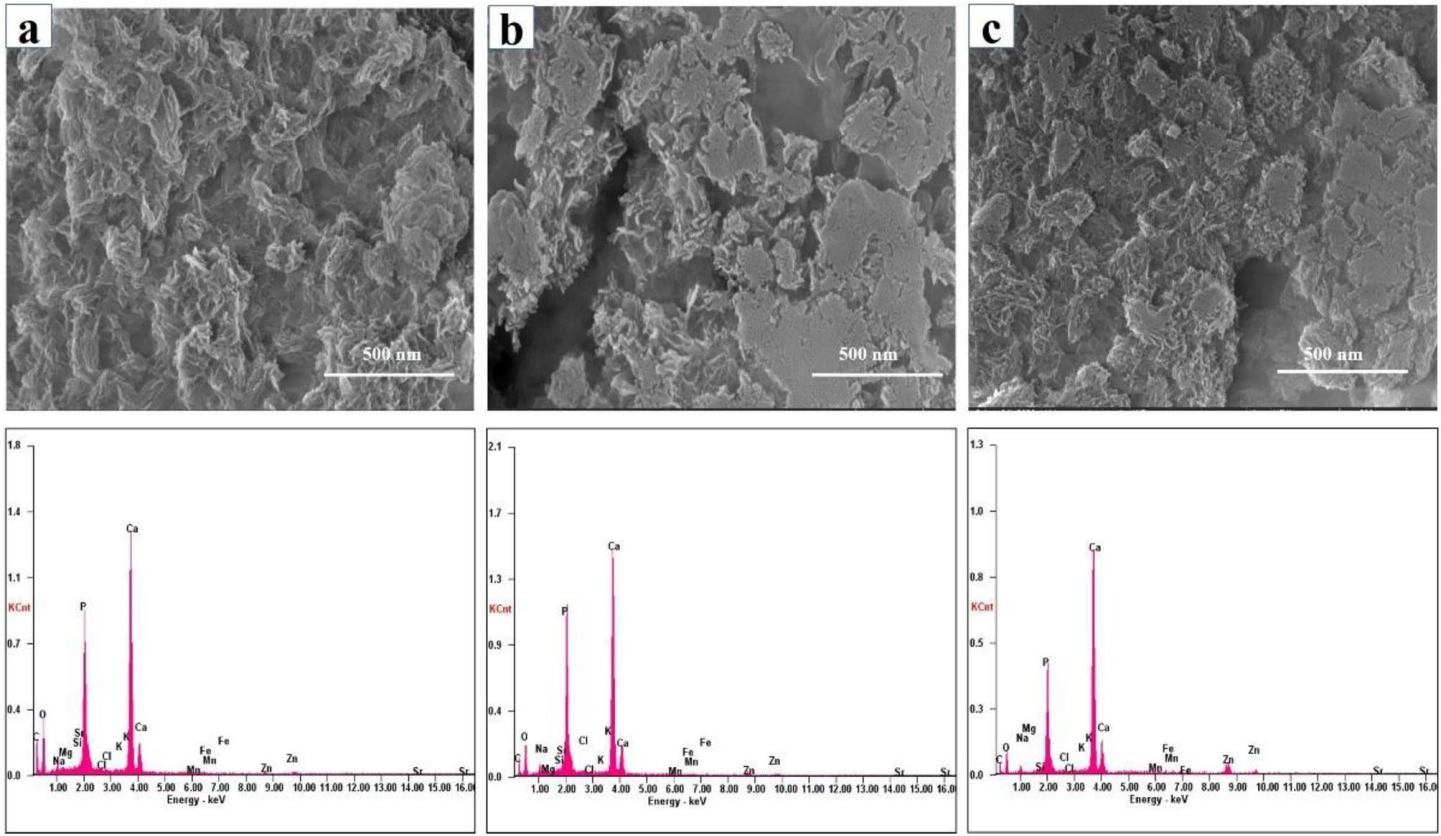
SEM–EDS analysis of (**a**) 2%Zn (**b**) 4% Zn (**c**) 6% SHA.

**Figure 6:**
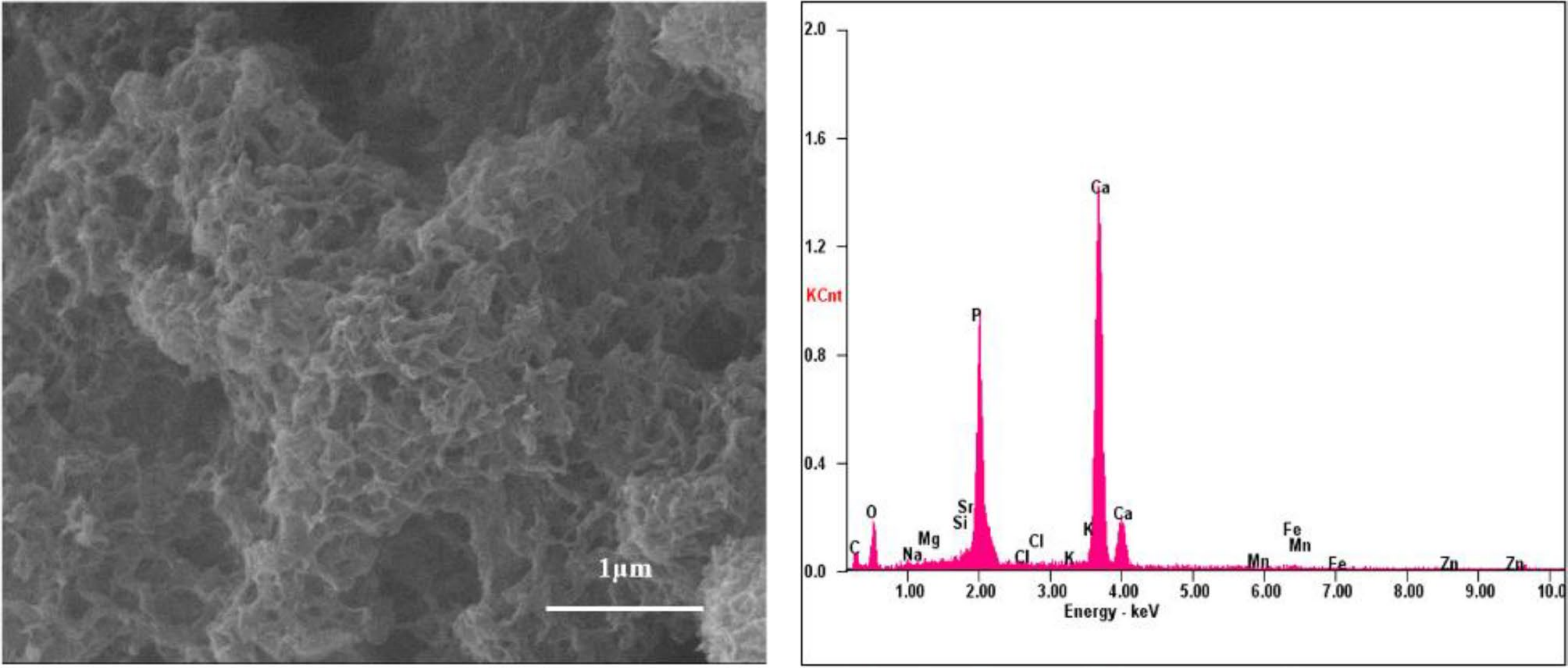
SEM analysis of 1% Sr, Zn, Mg SHA.

Largely agglomerated forms of HA nano crystals were illustrated in case of the SHA and substituted apatites (Figs. 3, 4, 5 and 6). Owing to very high surface area to volume ratio and higher surface energy, HA nanocrystals tend to agglomerate to quench the energy^41^. No discernible differences in the architecture of SHA and substituted SHA samples was observed with varying concentrations. The amount of Sr, Mg, and Zn substitutions researched in this study did not influence the particle morphology^5,27^. Suchanek et al. depicted similar acicular large agglomerated morphologies of Mg-HA, previously^27^. While Ren et al. described typical particle shapes without sharp face angles for Zn HA^43^. In all cases, SEM micrographs showed that the crystallites of samples were aggregated resulting in the formation of nano-sized particles and additionally larger agglomerates as well. Only slight decrease in size of apatite particles was witnessed owing to lower concentrations studied. Earlier, decreased particle size of HA was reported with increasing Zn fraction up to 17 mol%. This is indicative of the decreasing crystallinity of the apatite with increasing Zn fraction, as evidenced in XRD diffraction^44^.

Importantly, uniform agglomeration of irregular nanostructures could be observed which is known to differ based on co-doping ion chosen^3^. The surface of the 1% Sr, Zn, Mg SHA appeared to have a granular, nano-topography, likely to improve the overall porosity and surface area. This holds importance in optimization of the adsorption of specific proteins, which can support osteoblast cell adhesion and augment osteoblast proliferation and differentiation^26^.

EDS pattern of substituted HA showed increase in Mg, Sr and Zn ions upon comparison with SHA (Figs. 3, 4, 5 and 6). Morphologies and ion-substitution tendencies were consistent with the study by Cox et al.^5^. The increase in Sr, Zn and Mg ion% on SHA was confirmed by ICP-OES analysis (Table 3). Previously, bacterial synthesized HA co-substituted with Zn and Mg ions exhibited uniform agglomerated nanoparticles and EDS revealed successfully incorporation of 7.85 wt.% Zn and 0.49 wt.% Mg via the bacterial biomineralization method^12^. In the present study, the presence of other ions such as Na^+^, K^+^ and Cl^−^ ions owing to bacterial deposition was also seen as stated in the case of SHA and individual ion-substituted SHA. Thereby, HA synthesized using *Serratia sp* in both individual and co-substituted cases do not reflect any change in morphology or particle size yet clearly portrays incorporation of metallic ions in SHA lattice.

### TEM analysis

**Figure 7:**
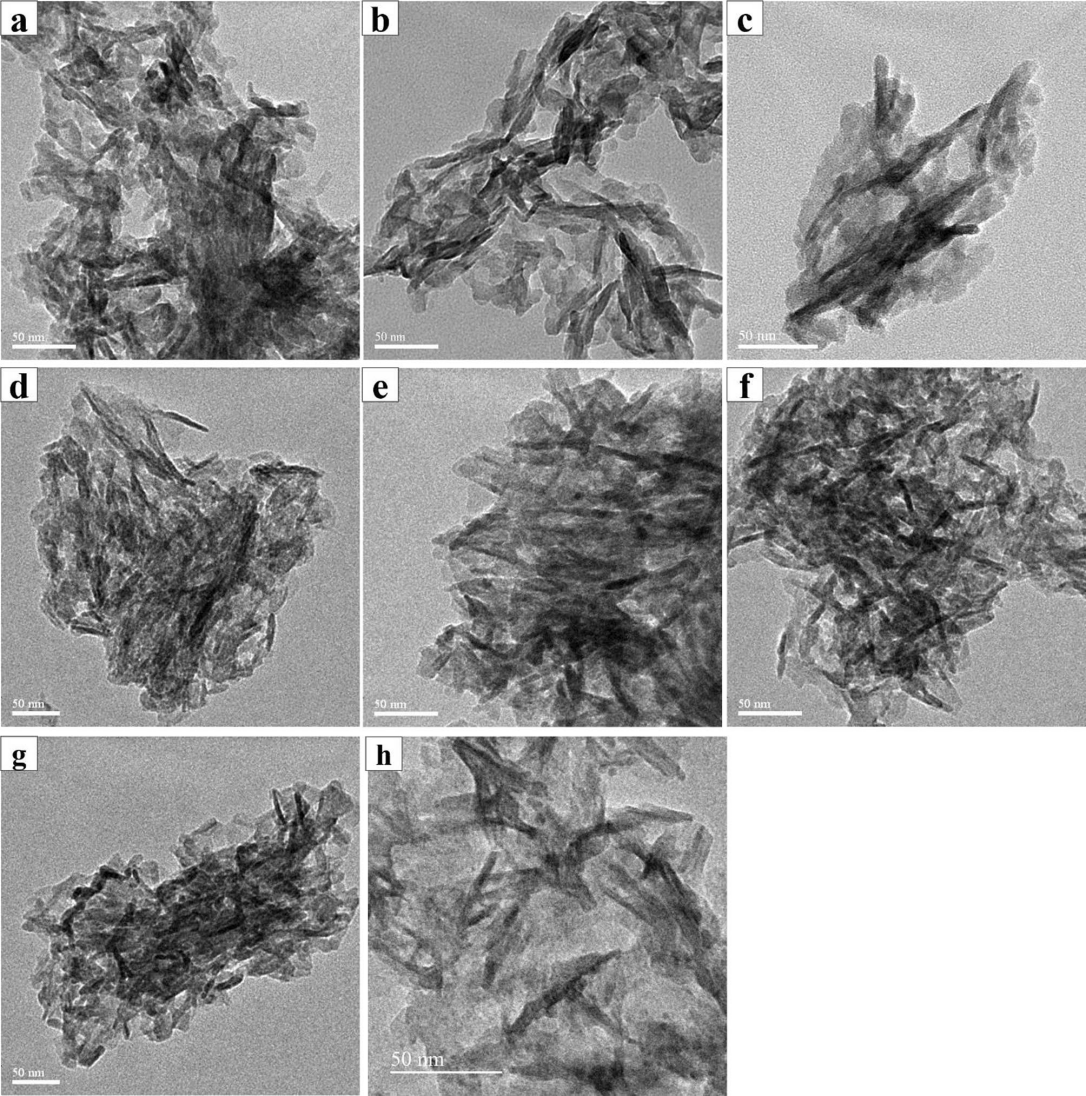
TEM analysis (**a**) 3% (**b**) 5% (**c**) 10% Sr SHA (**d**) 2% (**e**) 4% (**f**) 6% Zn SHA (**g**) 2% Mg SHA (**h**) SHA.

**Figure 8:**
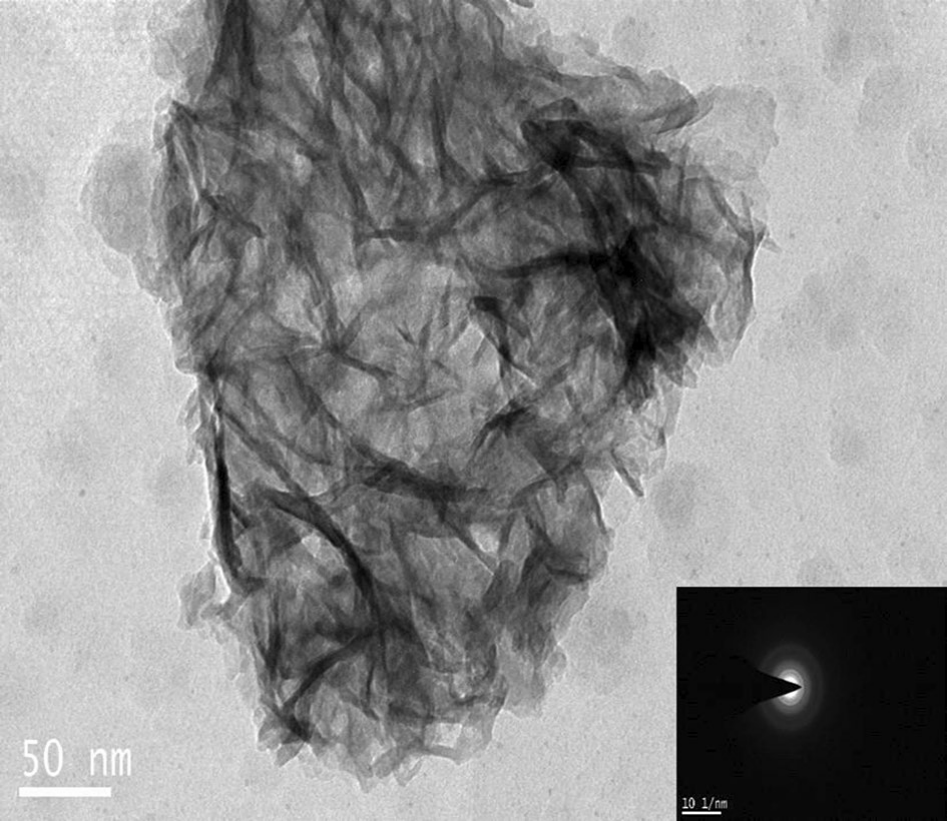
TEM analysis of 1% Sr, Zn, Mg SHA.

Figures 7a, b, c, d, e, f, g, h and 8 shows the morphology of SHA, individual and co-substituted SHA nanoparticles. Needle-like morphology was witnessed in SHA and all ion-substituted SHA. All particles were of nanoscale. The calculated average size of Sr SHA was around 20–50 nm of length and 8–15 nm of width which was slightly higher than SHA nanoparticles. While, in the case of Zn and Mg substituted SHA nanoparticles, the crystals were around 10–40 nm of length and 8–10 nm of width, almost similar to SHA nanoparticles. All synthesized and ion-substituted HA displayed nanorods and elongated shapes, such as needles. It could be seen that co-substitution of ions contributed to agglomeration tendencies of needle-like 1% Sr, Zn, Mg SHA. These nanoneedles build to form spherical nanoparticles that are interconnected with each other. The length of these needles or rods was between 40 and 70 nm and breadth between 4 and 10 nm. It can thus be assumed that the ion substitution influences the crystallinity and solubility of HA bearing superior biological performance in comparison to pure stoichiometric HA^28^.

Earlier, the Sr10%nHA and Zn10%HA substitutions were illustrated to be much smaller in size than nano HA with dimensions of 34.3 ± 4.2 nm; 9.6 ± 1.5 nm and 27.2 ± 6 nm; 7.6 ± 1.3 nm, respectively^26^. Our results were in alignment with reported typical lengths < 40 nm and breadths < 11 nm. Further, works have also indicated that substituted HA crystals tend to agglomerate as the concentration of the substituting ion increases. It was also observed that similar to reports that the nanocrystals for all of the ion-substitutions appeared granular in nature suggestive of high level of porosity of bacterial synthesized Sr, Zn and Mg SHA^26^.

Besides, needle-like nanoparticles of higher length 50–100 nm and width 10–20 nm in the case of Mg-Hap was reported for concentrations of 0.025, 0.05 and 0.1 M^3^. While, Ren et al. discussed the ultrastructure of ZnHA precipitates having agglomerated irregular flake-like shape^43^. Aggregated needle-shaped crystallites interconnected with fine rod-like particles was displayed by Li et al.^34^ of about 50–60 nm in length and width of 20 nm. The size of the particles was found to reduce with increasing Zn concentrations also indicated by XRD results. In all cases, size of the ion-substituted SHA exhibited smaller crystal dimensions as a result of bacterial deposition. In the human body, crystal size of apatite particle is reported as (5–20) × 60 nm. Hence, to a certain extent, the size of Sr, Zn and Mg substituted SHA resembles the natural bone parameters.

While, in the case of co-substitutions literature describes the average lengths of the particles of pure HA, 5Zn5Sr-HA, 5Zn10Sr-HA, 10Zn5Sr-HA, and 10Zn10Sr-HA were 68.4 ± 16, 39.4 ± 11.1, 47 ± 9.7, 27.5 ± 6.1 and 28.4 ± 5.8 nm respectively^42^. While, the TEM measurements for the nano-crystals of Sr5%/Zn5%nHA was 39.6 ± 5.8 nm in length and 11.0 ± 1.5 nm in breadth^26^. Besides, when Sr, Zn, Mg and Si were co-substituted on HA the length of needles or rods was between 20 and 50 nm, and their thickness was about 5 to 20 nm^28^. Herein, 1% Sr, Zn, Mg SHA (Fig. 8) presented dimensions in agreement with natural bone, a composite of organic collagen fibrils with inorganic, rod-shaped, nano-crystalline HA having an approximate length of 25–50 nm^26^. The size of particles was found to increase with ion-substitution of SHA lattice in substantial accord with XRD patterns (Table 2). Moreover, SAED reflected continuous ring patterns, validating that Sr^2+^, Zn^2+^ and Mg^2+^ co-substituted HA has a polycrystalline structure. The dimming of the SAED patterns indicated that the crystallinity of the 1% Sr, Zn, Mg SHA decreased, in line with results obtained via XRD and FTIR^42^.

**Table 2:** Structural data of 1% Sr, Zn, Mg SHA as compared to SHA.

Sample name	Lattice parameter (Å)		Cell volume (Å^3^)	Average crystallite size by XRD (nm)	Average crystallite size by TEM (L × B nm)	Crystallinity Index	Carbonate content (%)
	a	c					
SHA	9.41	6.86	526.4	9.6	Length- 20–50 Breadth- 5–10	0.019	3.13
1% Sr, Zn, Mg SHA	9.39	6.94	531.07	10.2	Length- 40–70 Breadth- 4–10	0.023	5.8

Overall, in the case of both individual ion-substitutions (Sr, Zn and Mg SHA) of varying concentrations and co-substitution (1% Sr, Zn, Mg SHA), XRD results clearly revealed crystallinity changes with addition of Sr, Zn and Mg ions. It is clear from prior studies that the substitutions of Zn^2+^ and Mg^2+^ decrease the lattice parameters, while Sr^2+^ increases the lattice parameters. Such ion substitutions influence the growth kinetics of c-plane of apatite in pseudo physiological solution and that they restrain the growth rate of c-plane through absorption at the kink sites of 2-dimensional island^42^. Further, the EDS results ascertained the presence of these ions in single apatite crystals. While, TEM results illustrated morphological changes in the HA crystals significantly with Sr addition^43^.

#### ICP-OES (ICP)

ICP-OES quantification revealed presence of trace elements in 1% Sr, Zn, Mg SHA and the Ca/P ratio was found to be 2.1 (Table 3). Mixed trends of increasing and decreasing Ca/P ratios have been reported. As the (Ca + Sr + Zn): P value reduced greatly with the addition of Zn^2+^ and Sr^2+^ and an even more significant decrease (from 1.83 to 1.58) by increasing the Zn^2+^ concentration up to 10% and Sr upto 5%. Thus reflecting formation of phosphates and inhibition of HA^42^. Similar decreasing values was obtained for the Sr-ZnHAp coatings including the Ca/P ratios of 1.60 ± 0.01, 1.38 ± 0.19, 1.16 ± 0.08 and 1.39 ± 0.12 for Hap, SrHAp, ZnHAp and Sr-ZnHAp, respectively (from 1.60 to 1.39). Besides, it is well recorded in literature that Ca/P theoretical value is 1.67.

**Table 3:** Comparison of ICP-OES of individual and co-substituted ions with SHA (%).

Elements	SHA	3%SrSHA	5%SrSHA	10%SrSHA	2%ZnSHA	4%ZnSHA	6%ZnSHA	2%MgSHA	1%Sr, Mg, Zn SHA
Ca	33.62	24.14	24.18	25.77	38.99	34.28	25.1	28.4	29.2
P	18.67	18.33	15.43	15.09	16.24	14.08	10.2	13.5	14.49
Na	1.12	0.91	1.01	1.38	0.9	0.89	0.67	1.05	0.83
Mg	0.19	0.39	0.26	0.38	0.37	0.37	0.13	0.59	0.38
Zn	0.05	0.18	0.1	0.02	1.21	2.3	2.5	0.018	0.49
Sr	0.04	0.18	0.27	0.5	0.02	0.02	0.013	0.018	0.68
K	0.03	0.08	0.08	0.07	0.09	0.08	0.03	0.055	0.063

In the case of bacterial mineralization Ca/P wt.% ratio of control HA was determined to be 1.97 and corresponding value for Zn-Mg-HA was 1.19^12^. Our results reveal an increased trend from 1.87 Ca/P value of SHA to 2.1 for 1% Sr, Zn, Mg SHA. It can further be stated that EDS measurements were good approximations for the elemental composition of nanocrystals of HA as estimated by ICP-OES. Moreover, the results obtained from FTIR was also in line with the Ca/P ratios. It is also worth highlighting, the agreement with SEM results with quantifiable substitution percentages of other cations such as Na^+^ and K^+^ ions. As mentioned, owing to bacterial deposition such rich deposition was observed and measured through ICP-OES that clearly validated the biomimetic multi-substituted hydroxyapatite.

It is also worthy to mention that individual Sr substitution decreased the Ca and P in 1% Sr, Zn, Mg SHA than SHA. However, substituting 2% Mg increased Ca and P deposition considerably. Progressive increase in Sr substitution increased its Sr content from 0.18% to highest value of 0.50%. Notably, upon comparison with the ion distribution in natural bone, (Table 3) SHA showed better comparison than 1% Sr, Zn, Mg SHA in terms of Ca and P.

Comparatively, 1% Sr, Zn, Mg SHA depicted higher deposition of Sr increasing from 0.04 to 0.68%, Zn from 0.05 to 0.49% and Mg from 0.19 to 0.38%. Thereby, co-substitution of all three ions together considerably increased Sr, Zn and Mg distribution of SHA. However, further research is required to optimize conditions for selectively increasing ion concentrations based on desired characteristics. It can be said that much lower levels of ion substitution is required in order to selectively enhance specific ion concentrations without affecting the yield of apatite.

#### Biocompatibility studies

**Figure 9:**
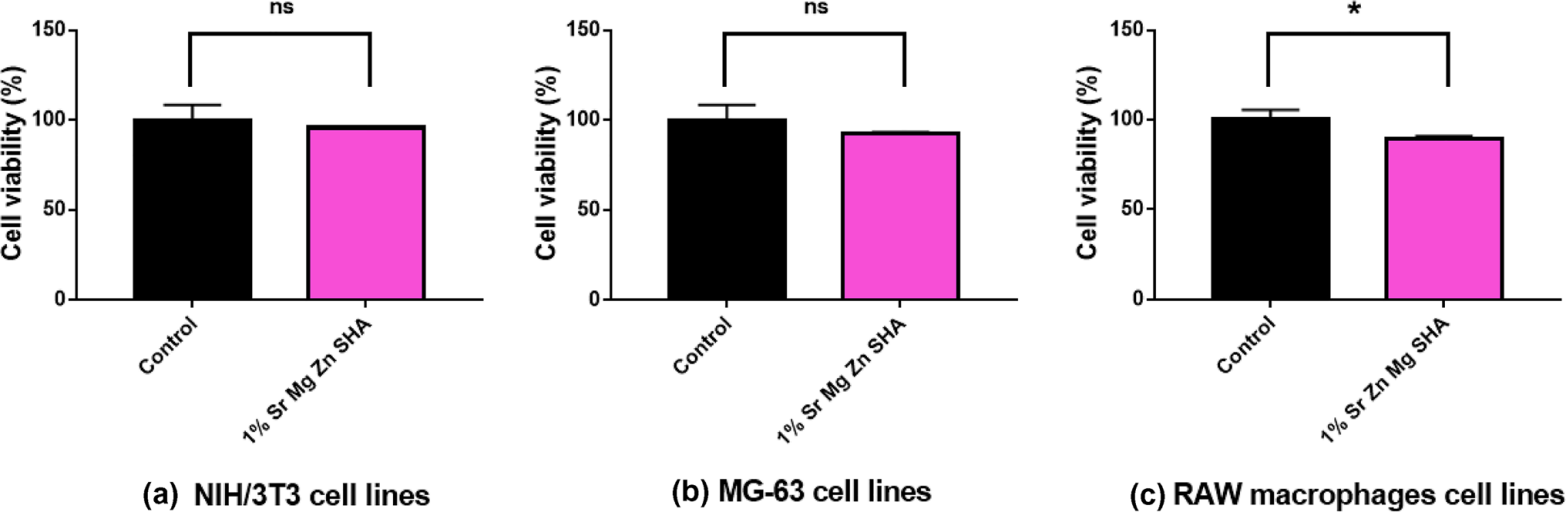
Cytotoxicity study of the synthesized 1% Sr, Mg, Zn SHA nanoparticles (**a**) NIH/3T3 cell lines (**b**) MG-63 osteosarcoma cell lines (**c**) RAW 264.7 macrophages cell lines n = 3; data shown as mean ± SD; Student T test, * *p* < 005).

Figure 9a, b and c depicts the biocompatibility of the 1% Sr, Zn, Mg SHA nanoparticles on NIH/3T3 fibroblast cells, MG-63 osteosarcoma cells and RAW 264.7 macrophages, respectively. 1% Sr, Zn, Mg SHA nanoparticles revealed nearly greater than 90% cell viability in all the cell lines studied. Cox et al. exploited the viability of MC3T3 cells on HAP, Mg-HAP, and Zn-HAP and reported no sign of cytotoxicity^5^. Furko et al. highlighted that the mHAp sample had the highest cell viability values with 85% after two days and 90% after two weeks compared to that of positive control^6^. The cell viability percentages obtained were 78 and 85% after 48 h, while after 2 weeks of culture the values were 81 and 90% on pure HAp and multi-ion modified HAp coatings, respectively. The present research exhibits (1% Sr, Zn, Mg SHA) a higher cell viability of for NIH/3T3, MG-63 and RAW 264.7 cell lines. It can be derived that co-substitution of ions augments the biocompatibility of sample. Additionally, it is well established that HA coatings facilitate the attachment and multiplication of osteoblastic cells as a result of its high hydrophilic property^6^.

Besides, de Lima et al. investigated the biocompatibility of various substituted HAP’s^45^. It was published that Mg-HAP and Zn-HAP enhanced the cell densities of murine fibroblasts. Importantly, the change in dissolution behaviour due to cationic substitution in place of Ca^2+^ and release of metal ions in the biological media; were labelled iconic factors in determining the biocompatibility of substituted HAP’s. Since the biocompatibility of substituted HAP’s is majorly determined by the dissolution behaviour of HAP’s and the extent of metal ions released from them, the crystallinity and level of substitution of ions is to be considered. XRD, FTIR and TEM analysis clearly depicted a reduction in crystallinity owing to the substitution of Sr^2+^, Zn^2+^ and Mg^2+^ ions in the HAP lattice. EDS analysis indicates a higher level of substitution of Sr^2+^, Zn^2+^ and Mg^2+^ in 1% Sr, Zn, Mg SHA confirmed by ICP-OES (Table 3). The solubility of HAP in biological medium is dependent on its crystallinity; there is an inverse proportionality of lower crystallinity, giving greater rates of dissolution^41^.

It is also important to note that the cell viability bears a strong dependence on the amount of substitution; with increased substitution, cell viability decreases. Sr concentrations in the range of 3–7 atom % significantly stimulate osteoblast activity and differentiation, as shown by the increased production of alkaline phosphatase activity, type I collagen and osteocalcin. Moreover, even at the lowest concentration, 1%, Sr affects osteoclast proliferation, which reduces with increasing Sr content^36^. It was shown that Mg^2+^ acts as an activator at optimum levels, however becomes inhibitory at very high concentrations. One possible explanation is that excess Mg^2+^ ions displaced Zn^2+^ from the catalytic site; since both metal ions could potentially bind to the same site^46^. While, the relatively decreased cell viability observed for co-substituted MgZnCo-HAP2 can be correlated to the greater amount of release of metal ions from it due to higher doping concentrations^41^.

Co-substitutions of ions thus are non-toxic and deliver significant advantages over single doping. Earlier, it was reported that Sr^2+^ and Zn^2+^ improves solubility, differentiation of osteoblasts, proliferation and cell adhesion of HA, hence the co-substitution of Sr^2^ and Zn^2+^ effectively enhanced the biological properties of HA. Furthermore, after 7 days of culturing, there was an increasing trend in the cell viability on co-substituted HA, confirming also the compatibility of all (Zn, Sr)-HA materials^47^. Moreover, Sr^2+^ interacted with calcium-sensitive receptors in pre-osteoblasts cell replication to activate Mitotic signal in the Wnt (Wingless/Integrated)/β-catenine signalling pathways supporting improved cell division^42^. While, Bodhak et al. investigated the in vitro bone cell–material interaction by seeding human fetal osteoblast cells on the Sr^2+^, Zn^2+^ and Mg^2+^ doped HAp surface up to 7 days. Enhanced cellular adherence and extracellular matrix formation were witnessed in the case of doped Hap, due to the advantageous surface properties and dopants chemistry. It was proposed that binary ions doped HA could be useful in the design of bone graft materials for HA based orthopaedic implants^48^.

Overall, the addition of Sr, Zn and Mg ions individually as well as co-substitutions to the bacterial solution maintained the presence of the HA structure (positions of XRD peaks and FTIR spectrum) but led to significant changes in its crystal dimensions (TEM) and crystallinity (XRD) due to the incorporation of the metal cations into the solid structure (Table 4). Until now, bacterial synthesized multi-ion co-substituted HA with possible applications for bone regeneration has not been studied. The present research demonstrated the feasibility of producing a bone graft substitute with highly beneficial properties for short time bone repair made of bacterial synthesized individual ion substituted and co-substituted HA. The aim of mimicking real bone composition was achieved using bacterial deposition and it can be clearly deduced that the synthesized SHA samples reflected similar characteristic traits of rich carbonated groups, low crystallinity, biocompatibility, nano-scale needle-shaped morphologies, dimensions, cell parameters and ion distributions. This research is believed to break apprehensions with regards to hydroxyapatite ion modifications and concentrations which were relatively less resorted or explored than property advantages associated with grain size, surface topology, morphology etc. The favourable aspects of osteoimmunodulatory and osteogenic capacities illustrate great potential for further research that could well inspire design of bioceramics for bone defect repair^49^.

**Table 4:** Comparison of individual and co-substituted ions in SHA.

	Individual	Co-substitution
FTIR	Characteristic HA groups (Phosphate, carbonate groups) present No broadening of OH bending or phosphate peaks Reduced crystallinity and high purity B-type carbonated HA Reduced intensity of peaks confirming ion incorporation	
XRD	No secondary phases No shifts Reduced crystallinity due to ion occupancy	
	Sr increased unit cell volume with Sr10% SHA of highest cell volume Zn and Mg SHA decreased cell volume 6% Zn SHA lattice decreased 2% Mg SHA lattice decreased	Decrease in a and increase in c lattice parameters Increased cell volume
SEM	Large agglomerated nanocrystals No difference in morphology for various concentrations and ions Uniform agglomeration EDS confirmed increase in ion % Na^+^, K^+^ and Cl^−^ ions present due to bacterial deposition	
TEM	Needle like nanoscale morphologies (Nanoneedles)	
	Sr SHA 20–50 nm length 8–15 nm width > SHA Zn SHA and Mg SHA 10–40 nm length and 8–10 nm width ≈ SHA	40–70 nm breadth and 4–10 nm > SHA

Extending from novelty of bacterial HA synthesis and ion co-substitutions, the economic and environmental aspects indeed need to be considered for future scope of scalability. Essentially, bacterial synthesis offered several advantages of simplicity, low processing temperatures, eco-friendly and highly pure products. Yet, measured understanding of the same is vital to understand the real-time environmental impact of producing such bioceramics and their inclined modifications. Furthermore, the control over complex multi-substituted HA synthesis and physicochemical properties is crucial towards the preparation of bio-minerals with improved biological response^28^. Besides, specific substitutions and co-substitutions provided information that could allow targeted healing approaches at defect sites. The vicarious ions incorporation would promote the SHA bioactivity and demonstrate osteoconductive/osteoinductive behaviours favouring therapeutic applications^3^. These outcomes reflect new verticals in using biologically synthesized nanoparticles for biomedical applications.

## Conclusion

*Serratia* biomineralized HA nanoparticles were substituted with individual as well as multiple ions at varying concentrations. Biomimetic synthesis approach was adopted which resulted in uniform agglomerates of SHA with carbonated groups, reduced crystallinity, devoid of secondary phases and nanoneedle morphologies. Analytical studies supported the symmetry of bone-like apatite to that of 1% Sr, Zn, Mg SHA. While, the changes in cell parameters and quantification of ions indicated successful substitution and co-substitution of select ions in the SHA lattice. Yet, results depicted that certain concentration of ion substitutions could become inhibitory as in the case of Mg SHA. Essentially, the ion substitutions and co-substitutions on SHA render the apatite nanoparticles similar to biological bone in terms of morphology, crystallinity, composition, dimensions, and biocompatibility. In terms of preliminary explorative research, the present study was confined to lower concentrations. However subsequent studies including a range of concentrations are required to be investigated. Further, the ability to deploy ion substituted and co-substituted biomineralized SHA for bone tissue engineering could greatly influence solubility and bio-functional parameters by appropriating optimum concentrations and combinations onto the SHA lattice.

## Data Availability

All data that support the plot within this paper and other finding of this study are available from the corresponding author on reasonable request.
